# Genomic analysis of codon usage shows influence of mutation pressure, natural selection, and host features on Marburg virus evolution

**DOI:** 10.1186/s12862-015-0456-4

**Published:** 2015-08-26

**Authors:** Izza Nasrullah, Azeem M Butt, Shifa Tahir, Muhammad Idrees, Yigang Tong

**Affiliations:** Department of Biochemistry, Faculty of Biological Sciences, Quaid-i-Azam University, Islamabad, 45320 Pakistan; Centre of Excellence in Molecular Biology (CEMB), University of the Punjab, Lahore, 53700 Pakistan; INRA, UMR85 Physiologie de la Reproduction et des Comportements, Nouzilly, F-37380 France; CNRS, UMR7247, F-37380 Nouzilly, France; Université François Rabelais de Tours, Tours, F-37380 France; State Key Laboratory of Pathogen and Biosecurity, Beijing Institute of Microbiology and Epidemiology, Beijing, 100071 People’s Republic of China

## Abstract

**Background:**

The Marburg virus (MARV) has a negative-sense single-stranded RNA genome, belongs to the family *Filoviridae*, and is responsible for several outbreaks of highly fatal hemorrhagic fever. Codon usage patterns of viruses reflect a series of evolutionary changes that enable viruses to shape their survival rates and fitness toward the external environment and, most importantly, their hosts. To understand the evolution of MARV at the codon level, we report a comprehensive analysis of synonymous codon usage patterns in MARV genomes. Multiple codon analysis approaches and statistical methods were performed to determine overall codon usage patterns, biases in codon usage, and influence of various factors, including mutation pressure, natural selection, and its two hosts, *Homo sapiens* and *Rousettus aegyptiacus*.

**Results:**

Nucleotide composition and relative synonymous codon usage (RSCU) analysis revealed that MARV shows mutation bias and prefers U- and A-ended codons to code amino acids. Effective number of codons analysis indicated that overall codon usage among MARV genomes is slightly biased. The Parity Rule 2 plot analysis showed that GC and AU nucleotides were not used proportionally which accounts for the presence of natural selection. Codon usage patterns of MARV were also found to be influenced by its hosts. This indicates that MARV have evolved codon usage patterns that are specific to both of its hosts. Moreover, selection pressure from *R. aegyptiacus* on the MARV RSCU patterns was found to be dominant compared with that from *H. sapiens*. Overall, mutation pressure was found to be the most important and dominant force that shapes codon usage patterns in MARV.

**Conclusions:**

To our knowledge, this is the first detailed codon usage analysis of MARV and extends our understanding of the mechanisms that contribute to codon usage and evolution of MARV.

**Electronic supplementary material:**

The online version of this article (doi:10.1186/s12862-015-0456-4) contains supplementary material, which is available to authorized users.

## Background

The Marburg virus (MARV) is a negative-sense single-stranded RNA virus with a genome size of 19 kb that encodes seven genes in a linear order. MARV belongs to family *Filoviridae*, which also includes the highly pathogenic Ebola virus (EBOV). The first documented evidence of MARV was in 1967 in laboratory workers and scientists at facilities in Germany and the former Yugoslavia via infected monkeys that were imported from north-western Uganda [1]. MARV is a zoonotic virus and has been detected in both infected and healthy Egyptian fruit bats (*Rousettus aegyptiacus*) in endemic areas in Africa; therefore, *R. aegyptiacus* are considered as its natural host. This is most likely the reason that MARV outbreaks have been mostly associated with individuals such as mine workers or tourists in the regions that these bats inhabit [2]. The typical symptoms include general malaise, acute fever, abdominal cramping, bleeding disorders, and shock [3]. Similar to its highly pathogenic cousin EBOV that is the cause of a recent ongoing outbreak, MARV also causes fatal viral hemorrhagic fever in humans and non-human primates with a fatality rate of up to 90 %. Therefore, there is a need for a detailed understanding of replication and evolution of this virus [3–5].

It is known that the genetic code shows redundancy and most of the amino acids can be translated by more than one codon. This redundancy represents a key step in modulating the efficiency and accuracy of protein production while maintaining the same amino acid sequence of the protein. Alternative codons within the same group that codes for the same amino acid are often termed ‘synonymous’ codons, although their corresponding tRNAs might differ in relative abundance in cells and in the speed by which they are recognized by the ribosome. However, the synonymous codons are not randomly chosen within and between genomes, which is referred to as codon usage bias [6, 7]. This phenomenon has been observed in a wide range of organisms, from prokaryotes to eukaryotes and viruses. Studies on codon usage have identified several factors that could influence codon usage patterns, including mutation pressure, natural or translational selection, secondary protein structure, replication and selective transcription, hydrophobicity and hydrophilicity of the protein, and the external environment [8–13]. Moreover, considering the virus’s genome size and other viral features, such as dependence on host’s machinery for key processes, including replication, protein synthesis, and transmission, compared with prokaryotic and eukaryotic genomes, the interplay of codon usage among viruses and their hosts is expected to affect overall viral survival, fitness, evasion from host’s immune system, and evolution [11, 14]. Therefore, knowledge of codon usage in viruses can reveal information about molecular evolution as well as improve our understanding of the regulation of viral gene expression and aid in vaccine design, where efficient expression of viral proteins may be required to generate immunity.

In the present study, we report genome-wide comprehensive analyses of codon usage and various factors that have contributed to the molecular evolution in MARV.

## Methods

### Analysis data

The complete genome sequences of 63 MARV strains were obtained from the National Center for Biotechnology (NCBI) GenBank database (http://www.ncbi.nlm.nih.gov). The accession numbers and demographics of the selected MARV genomes are provided in Additional file 1: Table S1.

### Recombination analysis

Identification of potential recombinant events in the MARV genomes were determined with the Recombination Detection Program (RDP) 4 Beta (version 4.27) software suite [15], which incorporates several phylogenetic-substitution and distance-based methods, including GENECONV [16], RDP [17], MaxChi [18], Chimaera [19], Bootscan [20], SiScan [21], 3Seq [22], and LARD [23]. The *P*-value cut-off was set to 0.05 in all analyses, and the Bonferroni correction was applied. The default settings were used for all analyses.

### Compositional analysis

The following compositional properties were calculated for the coding sequences of MARV genomes: (i) overall frequency of occurrence of the nucleotides (A%, C%, T/U%, and G%); (ii) frequency of each nucleotide at the third site of the synonymous codons (A_3S_%, C_3S_%, U_3S_%, and G_3S_%); (iii) frequencies of occurrence of nucleotides G + C at the first (GC_1S_), second (GC_2S_), and third synonymous codon positions (GC_3S_); (iv) mean frequencies of nucleotides G + C at the first and second position (GC_1,2S_); and (v) overall GC and AU content. The codons AUG and UGG are the only codons for Met and Trp, respectively, and the termination codons UAA, UAG, and UGA do not encode any amino acids. Therefore, these five codons are not expected to exhibit any usage bias and were therefore excluded from the analysis.

### Relative synonymous codon usage (RSCU) analysis

The RSCU values for all of the coding sequences of MARV genomes were calculated to determine the characteristics of synonymous codon usage without the confounding influence of amino acid composition and coding sequence size of different gene samples following a previously described method [24]. The RSCU index was calculated as follows:

$$ RSCU=\frac{g_{ij}}{{\displaystyle \sum_j^{n_i}{g}_{ij}}}{n}_i $$

where *g*_*ij*_ is the observed number of the *i*th codon for the *j*th amino acid, which has *n*_*i*_ kinds of synonymous codons. RSCU values represent the ratio between the observed usage frequency of one codon in a gene sample and the expected usage frequency in the synonymous codon family, given that all codons for the particular amino acid are used equally. The synonymous codons with RSCU values > 1.0 have positive codon usage bias and were defined as abundant codons, whereas those with RSCU values < 1.0 have negative codon usage bias and were defined as less-abundant codons. When the RSCU value is 1.0, it means there is no codon usage bias for that amino acid and the codons are chosen equally or randomly [25]. Moreover, the synonymous codons with RSCU values > 1.6 and < 0.6 were treated as over-represented and under-represented codons, respectively [26].

### Codon adaptation index (CAI) analysis

CAI analysis is a quantitative method that predicts the expression level of a gene based on its coding sequence. CAI values range from 0 to 1. The most frequent codons have the highest relative adaptiveness towards its host, and sequences with higher CAIs are suggested to be preferred over those with lower CAIs [27]. The CAI analysis for MARV genes was performed using CAIcal server [28]. The synonymous codon usage patterns of the viral hosts (*H. sapiens* and *R. aegyptiacus*) were used as references. Non-synonymous codons and termination codons were excluded from the calculation. The reference datasets for *H. sapiens* and *R. aegyptiacus* were obtained from the Codon Usage Database [29]. The correlation analysis between CAI and ENC values was performed to determine the relative influence of mutation and selection. If selection is preferred over mutation, the correlation (*r*) between the two quantities should be very high (*r* → −1). In contrast, if mutation force is more important, *r* should approach 0 (no correlation) [30, 31].

### Effective number of codons (ENC) analysis

ENC analysis was used to quantify the absolute codon usage bias by evaluating the degree of codon usage bias exhibited by the MARV coding sequences, regardless of gene length and the number of amino acids. ENC values range from 20, which indicates extreme codon usage bias using only one of the possible synonymous codons for the corresponding amino acid, to 61, which indicates no bias using all possible synonymous codons equally for the corresponding amino acid. The larger the extent of codon preference in a gene, the smaller the ENC value. It is also generally accepted that genes have a significant codon bias when the ENC value is less than or equal to 35 [32, 33]. ENC was calculated using the following formula:

$$ ENC=2+\frac{9}{{\overline{F}}_2}+\frac{1}{{\overline{F}}_3}+\frac{5}{{\overline{F}}_4}+\frac{3}{{\overline{F}}_6}, $$

Where ${\overline{F}}_k$ (*k* = 2, 3, 4, 6) is the mean of *F*_*k*_ values for the *k*-fold degenerate amino acids, which is estimated using the following formula:

$$ {F}_k=\frac{nS-1}{n-1}, $$

where *n* is the total number of occurrences of the codons for that amino acid and

$$ S={\displaystyle \sum_{i=1}^k{\left(\frac{n_i}{n}\right)}^2,} $$

where *n*_*i*_ is the total number of occurrences of the *i*th codon for that amino acid. Genes for which the codon choice is only constrained by a mutation bias will lie on or just below the curve of the expected ENC values. Therefore, to elucidate the relationship between GC_3S_ and ENC values, the expected ENC values for different GC_3S_ were calculated as follows:

$$ EN{C}^{expected}=2+s+\frac{29}{s^2+\left(1-{s}^2\right)}, $$

where *s* represents the given GC_3S_% [32].

### Principal component analysis (PCA)

PCA is a multivariate statistical method that is used to explore the relationships between variables and samples. In the present study, PCA was used to analyze the major trends in codon usage patterns among MARVs coding sequences. PCA involves a mathematical procedure that transforms correlated variables (RSCU values) into a smaller number of uncorrelated variables called principal components. To minimize the effect of amino acid composition on codon usage, each coding sequence was represented as a 59 dimensional vector, and each dimension corresponded to the RSCU value of each sense codon, which only included synonymous codons for a particular amino acid excluding the codons AUG, UGG, and the three stop codons.

### Neutral evolution analysis

The neutrality plot or neutral evolution analysis was performed to determine and compare the extent of influence of mutation pressure and natural selection on the codon usage patterns of MARV by plotting the *P*_12_ (GC_1,2S_) values of the synonymous codons against the *P*_3_ (GC_3S_) values. In the plot, the regression coefficient against *P*_3_ is regarded as the mutation–selection equilibrium coefficient and the evolutionary speed of the mutation pressure and natural selection pressure is expressed as the slope of a regression line. If all of the points lie along the diagonal distribution, no significant difference exists at the three codon positions, and there is no or weak external selection pressure. Alternatively, if the regression curve tends to be sloped or parallel to the horizontal axis, it means that the variation correlation between GC_1,2S_ and GC_3S_ is very low. Therefore, the regression curve effectively measures the degree of neutrality when selecting the effect that dominates evolution.

### Parity rule 2 (PR2) analysis

The Parity rule 2 (PR2) plot analysis was performed to investigate the impact of mutation and selection pressure on codon usage of genes. PR2 is a plot of AU-bias [A_3_/(A_3_ + U_3_)] as the ordinate and GC-bias [G_3_/(G_3_ + C_3_)] as the abscissa at the third codon position of the four-codon amino acids of entire genes. In this plot, the center of the plot, where both coordinates are 0.5, is the place where A = U and G = C (PR2), with no biasness between influence of mutation and selection rates (substitution rates) [34, 35].

### Influence of overall host codon usage on that of MARV

The RSCU values of MARVs and its two hosts, *H. sapiens* and *R. aegyptiacus*, were compared to determine influence of the host. The codon usage data of *H. sapiens* and *R. aegyptiacus* were obtained from the Codon Usage Database [29]. Furthermore, the influence of the overall codon usage patterns of hosts on the formation of the overall codon usage of viruses, defined as the similarity index *D*(*A*,*B*) [36], was calculated as follows:

$$ \begin{array}{l}R\left(A,B\right)=\frac{{\displaystyle \sum_{i=1}^{59}{a}_i\times {b}_i}}{\sqrt{{\displaystyle \sum_{i=1}^{59}{a}_i^2\times {b}_i^2}}}\\ {}D\left(A,B\right)=\frac{1=R\left(A,B\right)}{2},\end{array} $$

where *R*(*A*,*B*) is defined as a cosine value of an included angle between *A* and *B* spatial vectors and represents the degree of similarity between MARV and host overall codon usage pattern. *a*_*i*_ is defined as the RSCU value for a specific codon among 59 synonymous codons of MARV coding sequence. *b*_*i*_ is termed as the RSCU value for the same codon of the host. *D*(*A*,*B*) represents the potential effect of the overall codon usage of the host on that of MARV, and its value ranges from 0 to 1.0 [36].

### Relative dinucleotide abundance analysis

The relative abundance of dinucleotides in the coding regions of MARV genomes was calculated using a previously described method [37]. A comparison of actual and expected dinucleotide frequencies of the 16 dinucleotides in coding regions of MARV was also undertaken. The odds ratio was calculated using the following formula:

$$ {P}_{xy}=\frac{f_{xy}}{f_y{f}_x}, $$

where *f*_x_ denotes the frequency of nucleotide X, *f*_y_ denotes the frequency of nucleotide Y, *f*_y_*f*_x_ denotes the expected frequency of the dinucleotide XY, and *f*_xy_ the frequency of the dinucleotide XY. This was calculated for each dinucleotide. As a conservative criterion, for *P*_*xy*_ > 1.23 or < 0.78, the XY pair is considered to be over-represented or under-represented, respectively, in terms of relative abundance compared with a random association of mononucleotides.

### Correlation analysis

Correlation analysis was carried out to identify the relationships between nucleotide composition, PCA, and codon usage patterns of MARV using Spearman’s rank correlation analysis. All statistical analyses were carried out using SPSS 17 (SPSS Inc., Chicago, IL, USA) for Windows.

## Results

### Recombination analysis

It has been previously shown that occurrence of recombination events at either gene or genome levels can influence codon usage bias patterns. For example, recombination can influence the effect of natural selection on codon usage [38–41]. Therefore, to avoid influence of recombination on codon analysis, we first performed recombination analysis on the 63 MARV genomes. No evidence of recombination was found among MARV genomes. Therefore, coding sequences of all 63 of the initially selected MARV genomes were included in codon usage analysis as discussed in following sections.

### MARV coding sequences are enriched with A and U nucleotides

To determine the potential impact of nucleotide constraints on codon usage, nucleotide composition analysis was performed. It was found that the A and U nucleotides were most abundant in MARV coding sequences with a mean composition of 31.9 and 27.7 %, respectively, compared with C (20.8 %) and G (19.6 %). The nucleotide composition at the third position of synonymous codons (A_3S_, U_3S_, G_3S_, C_3S_) showed that the mean A_3S_ (31.3 %) and U_3S_ (33.0 %) were also highest compared with G_3S_ (17.7 %) and C_3S_ (18 %) (Table 1). The mean AU and GC compositions were determined to be 59.6 and 40.4 %, respectively, highlighting that there is an AU-rich composition of MARV coding sequences.

**Table 1:** Nucleotide composition analysis of MARV coding sequences (%)

No	A	U	G	C	A_3S_	U_3S_	G_3S_	C_3S_	AU	GC	GC_1S_	GC_2S_	AU_3S_	GC_3S_	GC_12S_	ENC	ARO	GRAVY
1	31.9	27.6	19.7	20.8	31.2	32.8	18.1	18	59.5	40.5	46.4	39	64	36.1	42.7	54.76	9	−32
2	32	27.8	19.5	20.7	31.6	33.3	17.4	17.8	59.8	40.2	46.5	38.9	64.9	35.2	42.7	54.96	9	−33
3	32	27.8	19.5	20.6	31.5	33.4	17.4	17.8	59.8	40.2	46.5	38.9	64.9	35.1	42.7	53.54	9	−32
4	32	27.7	19.5	20.7	31.5	33.2	17.4	17.9	59.7	40.3	46.5	38.9	64.7	35.3	42.7	53.59	9	−33
5	32	27.7	19.6	20.8	31.5	33.2	17.4	18	59.7	40.3	46.6	39	64.7	35.3	42.8	53.59	9	−33
6	32	27.8	19.5	20.6	31.5	33.4	17.4	17.8	59.8	40.2	46.5	38.9	64.9	35.1	42.7	53.57	9	−32
7	32	27.6	19.5	20.9	31.4	33	17.5	18.1	59.6	40.4	46.4	39	64.4	35.6	42.7	53.66	9	−32
8	32	27.7	19.5	20.8	31.4	33	17.6	18	59.7	40.4	46.5	39.1	64.4	35.6	42.8	53.64	9	−33
9	32	27.8	19.6	20.6	31.5	33.4	17.3	17.7	59.8	40.2	46.6	38.9	64.9	35.1	42.75	54.67	9	−33
10	32	27.6	19.5	20.9	31.4	32.9	17.5	18.2	59.6	40.4	46.5	39.1	64.3	35.7	42.8	54.17	9	−33
11	31.9	27.5	19.7	20.9	31.2	32.6	18	18.2	59.4	40.6	46.6	38.8	63.8	36.3	42.7	53.43	9	−32
12	31.9	27.7	19.6	20.8	31.2	33.2	17.7	17.9	59.6	40.4	46.4	39.1	64.4	35.6	42.75	54.64	9	−33
13	31.9	27.7	19.6	20.8	31.2	33.2	17.6	17.9	59.6	40.3	46.4	39.1	64.4	35.5	42.75	54.47	9	−33
14	32	27.7	19.5	20.8	31.4	33	17.6	18	59.7	40.4	46.5	39.1	64.4	35.6	42.8	54.29	9	−33
15	32	27.7	19.5	20.8	31.4	33	17.5	18	59.7	40.3	46.4	39	64.4	35.5	42.7	54.14	9	−33
16	32	27.6	19.5	20.8	31.4	33	17.5	18.1	59.6	40.3	46.4	39	64.4	35.6	42.7	53.91	9	−33
17	32	27.7	19.5	20.8	31.3	33.1	17.6	18	59.7	40.4	46.5	39.1	64.4	35.6	42.8	54.31	9	−33
18	32	27.6	19.5	20.8	31.4	33	17.5	18	59.6	40.3	46.4	39	64.4	35.6	42.7	54.36	9	−33
19	32	27.7	19.5	20.8	31.4	33	17.5	18	59.7	40.3	46.4	39	64.4	35.5	42.7	54.31	9	−33
20	32	27.7	19.5	20.8	31.4	33	17.5	18	59.7	40.3	46.4	39	64.4	35.5	42.7	54.33	9	−33
21	32	27.6	19.5	20.8	31.4	33	17.5	18.1	59.6	40.3	46.4	39	64.4	35.6	42.7	54.31	9	−33
22	32	27.7	19.5	20.8	31.3	33.2	17.6	17.9	59.7	40.3	46.5	39.1	64.5	35.5	42.8	54.39	9	−33
23	32	27.6	19.5	20.8	31.4	33	17.5	18	59.6	40.3	46.4	39	64.4	35.5	42.7	54.27	9	−33
24	32	27.6	19.5	20.9	31.4	33	17.5	18.1	59.6	40.3	46.4	39	64.4	35.6	42.7	54.34	9	−33
25	32	27.6	19.5	20.9	31.4	33	17.5	18.1	59.6	40.4	46.5	39	64.4	35.6	42.75	54.30	9	−33
26	32	27.7	19.5	20.9	31.3	32.9	17.7	18.1	59.7	40.4	46.4	39	64.2	35.8	42.7	54.63	9	−33
27	32	27.7	19.5	20.9	31.3	32.9	17.7	18.1	59.7	40.4	46.4	39	64.2	35.8	42.7	54.63	9	−33
28	32	27.6	19.5	20.9	31.3	33	17.6	18.1	59.6	40.3	46.3	39	64.3	35.7	42.65	54.37	9	−33
29	32	27.6	19.5	20.9	31.4	33	17.5	18.1	59.6	40.3	46.4	39	64.4	35.6	42.7	53.96	9	−33
30	32	27.6	19.5	20.9	31.4	33	17.5	18.1	59.6	40.4	46.4	39.1	64.4	35.6	42.75	54.26	9	−33
31	32	27.6	19.5	20.9	31.4	33	17.5	18.1	59.6	40.4	46.5	39	64.4	35.6	42.75	54.40	9	−33
32	32	27.6	19.5	20.9	31.4	33	17.5	18.1	59.6	40.4	46.4	39	64.4	35.6	42.7	54.29	9	−33
33	32	27.6	19.5	20.9	31.3	33	17.6	18.1	59.6	40.4	46.5	39	64.3	35.7	42.75	54.26	9	−33
34	32	27.7	19.5	20.8	31.3	33.2	17.6	17.9	59.7	40.3	46.4	39.1	64.5	35.5	42.75	54.76	9	−33
35	32	27.6	19.5	20.9	31.4	33	17.5	18	59.6	40.4	46.5	39	64.4	35.6	42.75	54.24	9	−33
36	32	27.7	19.5	20.8	31.3	33.2	17.6	17.9	59.7	40.3	46.4	39.1	64.5	35.5	42.75	53.86	9	−33
37	31.7	27.7	19.7	20.9	30.8	33	18.1	18.1	59.4	40.6	46.7	38.9	63.8	36.2	42.8	54.01	9	−32
38	31.7	27.7	19.7	20.9	30.8	33	18.1	18.1	59.4	40.6	46.7	38.9	63.8	36.2	42.8	54.30	9	−32
39	31.7	27.7	19.7	20.9	30.8	33	18.1	18.1	59.4	40.6	46.7	38.9	63.8	36.2	42.8	54.20	9	−32
40	31.7	27.6	19.7	20.9	30.8	33	18.1	18.1	59.3	40.6	46.7	38.9	63.8	36.2	42.8	54.11	9	−32
41	31.7	27.7	19.7	20.9	30.8	33	18.1	18.1	59.4	40.6	46.7	38.9	63.8	36.2	42.8	54.26	9	−32
42	31.7	27.7	19.7	20.9	30.8	33	18.1	18.1	59.4	40.6	46.7	38.9	63.8	36.2	42.8	54.24	9	−32
43	31.7	27.7	19.7	20.9	30.8	33	18.1	18.1	59.4	40.6	46.7	38.9	63.8	36.2	42.8	54.20	9	−32
44	31.7	27.7	19.7	20.9	30.8	33	18.1	18.1	59.4	40.6	46.7	38.9	63.8	36.2	42.8	54.19	9	−32
45	32	27.7	19.5	20.8	31.5	33.1	17.5	18	59.7	40.3	46.4	39.1	64.6	35.4	42.75	54.21	9	−33
46	32	27.9	19.6	20.5	31.4	33.5	17.5	17.6	59.9	40.1	46.3	38.9	64.9	35.1	42.6	54.24	9	−33
47	32	27.9	19.6	20.6	31.4	33.5	17.5	17.6	59.9	40.1	46.3	38.9	64.9	35.1	42.6	54.23	9	−33
48	32	27.7	19.5	20.8	31.4	33.1	17.5	18	59.7	40.3	46.4	39	64.5	35.5	42.7	54.46	9	−33
49	32	27.7	19.5	20.8	31.4	33	17.5	18.1	59.7	40.3	46.4	39	64.4	35.6	42.7	53.63	9	−33
50	32	27.9	19.6	20.6	31.4	33.4	17.5	17.6	59.9	40.1	46.3	38.9	64.8	35.2	42.6	53.40	9	−33
51	32	27.6	19.6	20.9	31.4	32.7	17.8	18.1	59.6	40.4	46.5	38.9	64.1	35.8	42.7	54.03	9	32
52	31.9	27.5	19.6	20.9	31.2	32.6	18	18.2	59.4	40.6	46.5	39	63.8	36.2	42.75	54.09	9	−33
53	32	27.7	19.5	20.8	31.4	33.1	17.5	17.9	59.7	40.3	46.5	39.1	64.5	35.5	42.8	53.60	9	−33
54	32	27.8	19.5	20.6	31.6	33.3	17.3	17.9	59.8	40.2	46.4	39	64.9	35.1	42.7	54.46	9	−33
55	32	27.7	19.5	20.8	31.3	33.1	17.6	18	59.7	40.4	46.4	39	64.4	35.6	42.7	54.47	9	−33
56	31.9	27.5	19.6	21	31.2	32.4	18	18.4	59.4	40.6	46.6	38.8	63.6	36.3	42.7	53.53	9	−32
57	32	27.6	19.5	20.8	31.4	33.1	17.6	18	59.6	40.3	46.4	39.1	64.5	35.6	42.75	54.56	9	−33
58	32	27.6	19.5	20.9	31.4	33	17.5	18.1	59.6	40.4	46.5	39	64.4	35.6	42.75	54.09	9	−33
59	32.1	27.8	19.5	20.6	31.6	33.4	17.3	17.8	59.9	40.1	46.4	38.9	65	35.1	42.65	54.17	9	−33
60	32	27.4	19.6	21	31.4	32.4	17.9	18.4	59.4	40.6	46.6	38.9	63.8	36.2	42.75	54.44	9	−33
61	32	27.4	19.6	21	31.3	32.4	17.9	18.4	59.4	40.6	46.6	38.9	63.7	36.3	42.75	53.87	9	−33
62	31.8	27.6	19.7	20.9	30.9	32.9	18.1	18.1	59.4	40.6	46.7	38.9	63.8	36.2	42.8	54.37	9	−32
63	31.9	27.6	19.6	20.9	31.1	33	17.8	18.1	59.5	40.5	46.5	39.1	64.1	35.9	42.8	54.33	9	−33
Avg	31.9	27.7	19.6	20.8	31.3	33.0	17.7	18.0	59.6	40.4	46.5	38.9	64.3	35.7	42.7	54.20	9	−.30
SD	0.1	0.1	0.08	0.11	0.22	0.23	0.25	0.16	0.15	0.15	0.12	0.08	0.36	0.36	0.05	0.35	0	0.004

*ENC* effective number of codons; *GRAVY* general average hydropathicity; *ARO* aromaticity, *Avg* average; *SD* standard deviation

The analysis of nucleotide composition at first, second, and third positions of synonymous codons showed that GC_1S_ values ranged from 46.3 to 46.7 %, with a mean of 46.5 % and standard deviation (SD) of 0.12. GC_2S_ values ranged from 38.8 to 39.1%, with a mean of 39.0 % and an SD 0.08. GC_1,2S_ values ranged from 42.6 to 42.8 %, with an average of 42.7 % and SD of 0.05. In the case of GC_3S_, the values ranged from 35.1 to 36.3 %, with a mean of 35.67 % and SD of 0.36; alternatively, the AU_3S_ values ranged from 63.6 to 65.0 %, with a mean of 64.33 % and an SD of 0.36. These data confirmed that a substantial portion of MARV coding sequences are composed of A and U nucleotides (Table 1).

### A- and U-ended codons are preferred in MARV coding sequences

The patterns of synonymous codon usage in MARV coding sequences were assessed by RSCU analysis. All of the 18 most abundantly used codons for their corresponding amino acids in MARV coding sequences were A/U- ended and exhibited an equal distribution of A and U (A-ended: 9; U-ended: 9) (Table 2, Additional file 2: Figure S1). Analysis of over- and under-representation of codons showed that four out of 18 preferred codons had RSCU values >1.6. These are UUA(L), ACA(T), UCA(S), and AGA(R), whereas the RSCU values of the remaining preferred codons were also found to be >0.6 and <1.6. However, the under-represented (RSCU <0.6) and non-preferred codons were all G/C-ended (Table 2). Nucleotide composition and RSCU analyses showed that selection of the preferred codons has been mostly influenced by compositional constraints (A and U in this case), which accounts for the presence of mutation pressure.

**Table 2:** The synonymous codon usage patterns of MARV and its hosts

Codon (Amino acid)	RSCU			Codon (Amino acid)	RSCU		
	MARV	*H. sapiens*	*R. aegyptiacus*		MARV	*H. sapiens*	*R. aegyptiacus*
UUU (F)	**1.23**	0.92	0.68	UCU (S)	1.25	1.14	0.72
UUC (F)	0.77	**1.08**	**1.32**	UCC (S)	0.66	1.32	1.62
UUA (L)	**1.72**	0.48	0.18	UCA (S)	**1.80**	0.90	0.48
UUG (L)	1.24	0.78	0.78	UCG (S)	0.28	0.30	0.30
CUU (L)	1.08	0.78	0.72	AGU (S)	1.28	0.90	0.72
CUC (L)	0.64	1.20	1.50	AGC (S)	0.73	**1.44**	**2.16**
CUA (L)	0.80	0.42	0.30	AGA (R)	**2.00**	**1.26**	1.80
CUG (L)	0.53	**2.40**	**2.52**	CGU (R)	0.82	0.48	0.00
AUU (I)	**1.35**	1.08	0.78	CGC (R)	0.34	1.08	0.66
AUC (I)	0.73	**1.41**	**1.86**	CGA (R)	0.94	0.66	0.66
AUA (I)	0.92	0.51	0.36	CGG (R)	0.55	1.20	0.66
GUU (V)	**1.38**	0.72	0.36	AGG (R)	1.46	**1.26**	**2.16**
GUC (V)	0.96	0.96	0.92	UGU (C)	**1.39**	0.92	0.76
GUA (V)	0.80	0.48	0.28	UGC (C)	0.61	**1.08**	**1.24**
GUG (V)	0.86	**1.84**	**2.44**	CAU (H)	**1.37**	0.84	0.58
CCU (P)	**1.51**	1.16	**1.56**	CAC (H)	0.63	**1.16**	**1.42**
CCC (P)	0.77	**1.28**	1.16	CAA (Q)	**1.35**	0.54	0.58
CCA (P)	1.26	1.12	0.72	CAG (Q)	0.65	**1.46**	**1.42**
CCG (P)	0.46	0.44	0.56	AAU (N)	**1.35**	0.94	0.50
ACU (T)	1.21	1.00	1.04	AAC (N)	0.65	**1.06**	**1.50**
ACC (T)	0.62	**1.44**	**1.72**	AAA (K)	**1.24**	0.86	0.54
ACA (T)	**1.85**	1.12	1.12	AAG (K)	0.76	**1.14**	**1.46**
ACG (T)	0.33	0.44	0.08	GAU (D)	**1.25**	0.92	0.56
GCU (A)	1.37	1.08	1.04	GAC (D)	0.75	**1.08**	**1.44**
GCC (A)	0.90	**1.60**	**1.48**	GAA (E)	**1.31**	0.84	0.62
GCA (A)	**1.47**	0.92	0.84	GAG (E)	0.69	**1.16**	**1.38**
GCG (A)	0.27	0.44	0.64	GGU (G)	1.03	0.64	0.84
UAU (Y)	**1.31**	0.88	0.66	GGC (G)	0.52	**1.36**	0.84
UAC (Y)	0.69	**1.12**	**1.34**	GGA (G)	**1.43**	1.00	**1.20**
				GGG (G)	1.01	1.00	1.12

Preferred codons are shown in bold

### Intra-genes codon usage bias is low in MARV

To estimate the degree of codon usage bias within coding sequences of different isolates of MARV, ENC were calculated. The ENC values among MARV coding sequences ranged from 53.4 to 54.9, with a mean of 54.2 (ENC > 40) and an SD of 0.35 (Table 1), indicating a relatively stable and conserved genomic composition among different MARV coding sequences.

### Trends in codon usage variation

**Fig. 1:**
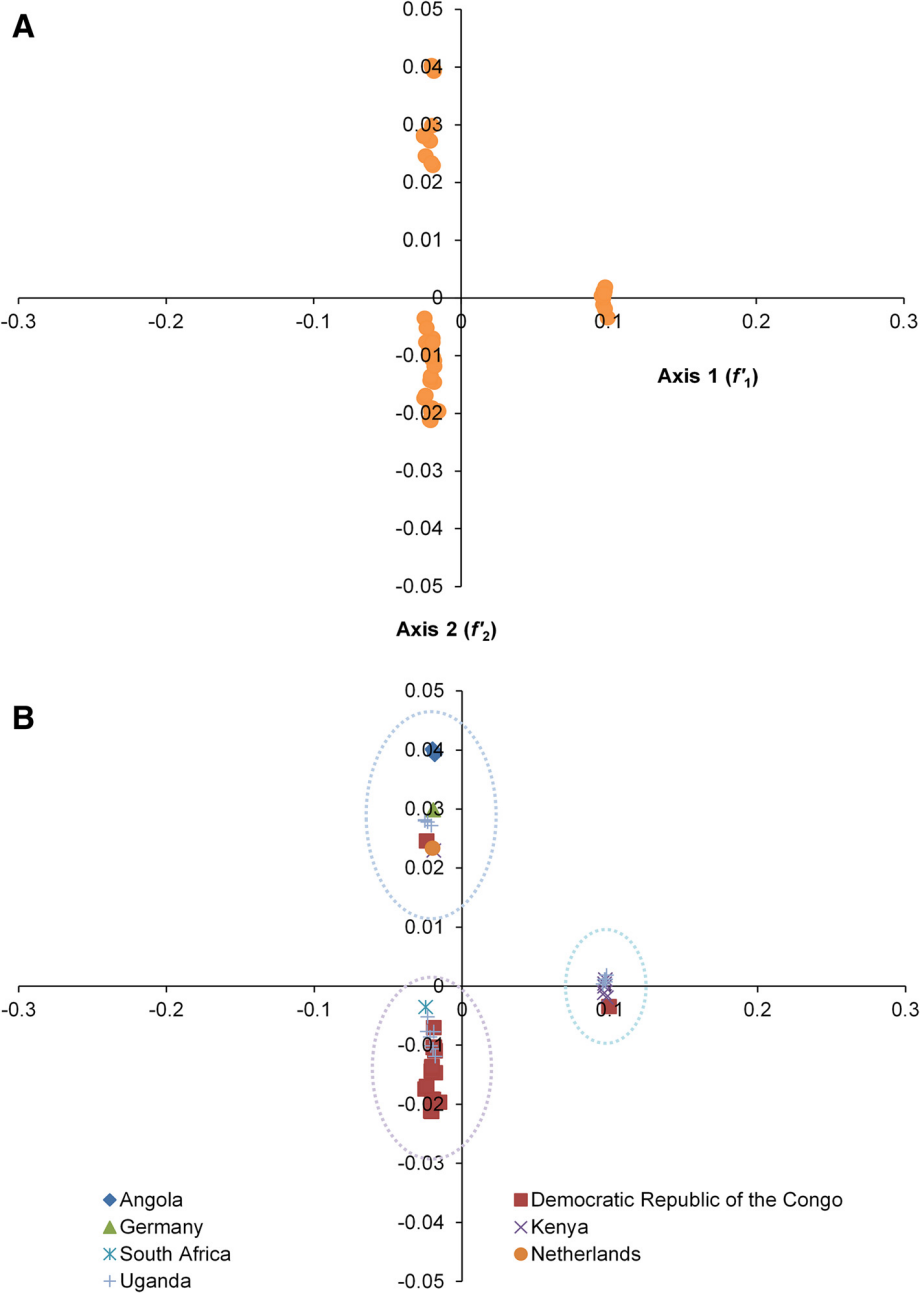
**a** Correspondence analysis of codon usage patterns in MARV coding sequences. **b** Correspondence analysis of codon usage patterns in MARV coding sequences based on region of isolation

To determine the trends in codon usage variation among coding sequences of different MARV isolates, we performed PCA on the RSCU values, which were examined as a single dataset (Fig. 1a). The first principal axis (*f'*_1_) accounted for 65.55 % of the total variation, and the next three axes (*f'*_2_-*f'*_4_) accounted for 14.17, 10.48, and 2.36 % of the total variation in synonymous codon usage, respectively. Next, we plotted principal axes based on geographical locations of MARV isolates (Fig. 1b). Three separate clusters were observed. Cluster A, which formed the largest cluster, consisted of isolates from the Demographic Republic of Congo (DRC), Uganda, and a single isolate from South Africa. The DRC isolates formed the majority of Cluster A. Cluster B was dominated by isolates from Uganda, Kenya, and a single isolate from DRC, whereas Cluster C consisted of all of the isolates from Angola and single isolates from Germany, the Netherlands, Uganda, and DRC.

### PR2 biasness analysis

**Fig. 2:**
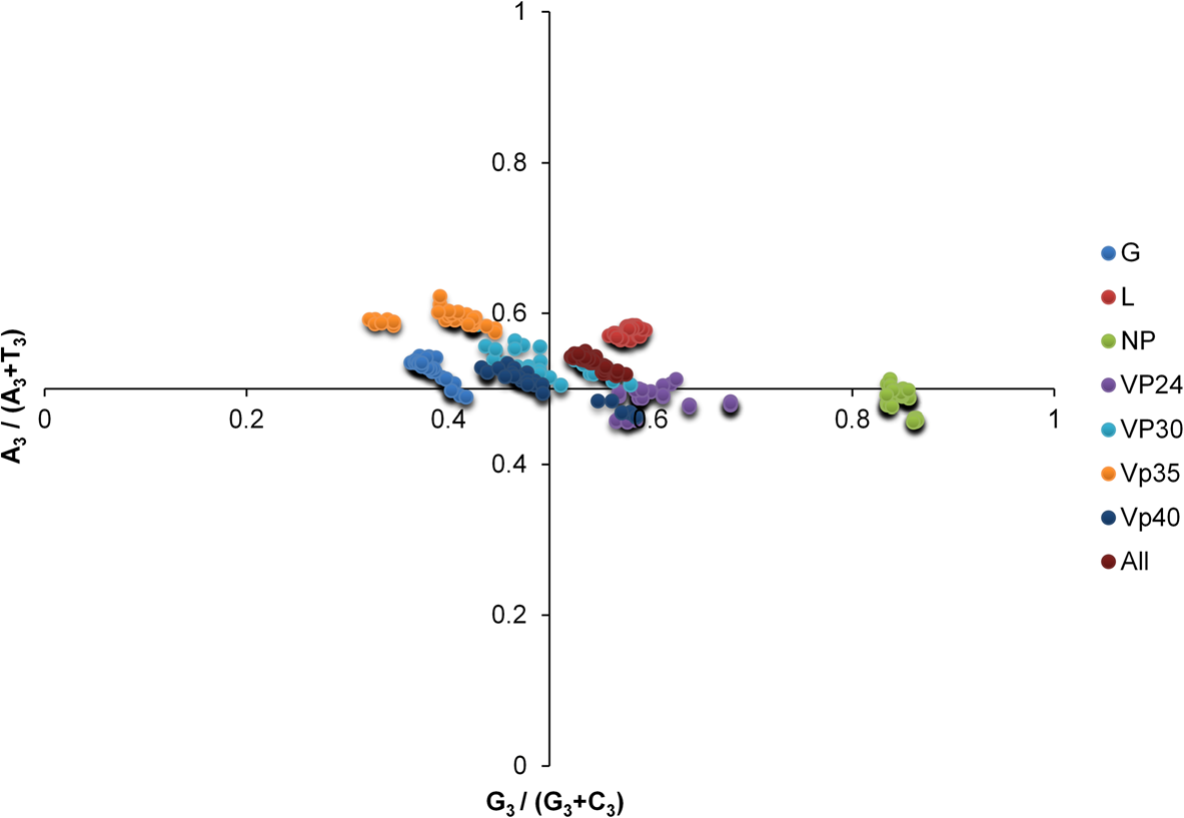
Parity Rule 2 (PR2)-bias plot [A_3_/(A_3_ + U_3_) against G_3_/(G_3_+ C_3_)]. PR2 biasness plot is calculated for whole genome and individual MARVs genes which are indicated by “All” and one letter codes respectively. G: Glycoprotein; NP: Nucleoprotein; L: RNA-directed RNA polymerase; VP24: Membrane associated protein; VP40: matrix protein

To determine whether the biased codon choices are restricted to highly biased genes, the relation between A and U content and G and C content in four-fold degenerate codon families (alanine, arginine, glycine, leucine, proline, serine, threonine and valine) were analyzed by PR2 plot (Fig. 2). It was found that A and U were used more frequently than G and C in MARV four fold degenerate codon families. This shows that preference towards codon choices are shaped by both mutation pressure and other factors including natural selection.

### Mutation pressure dominates shaping of MARV codon usage patterns

**Fig. 3:**
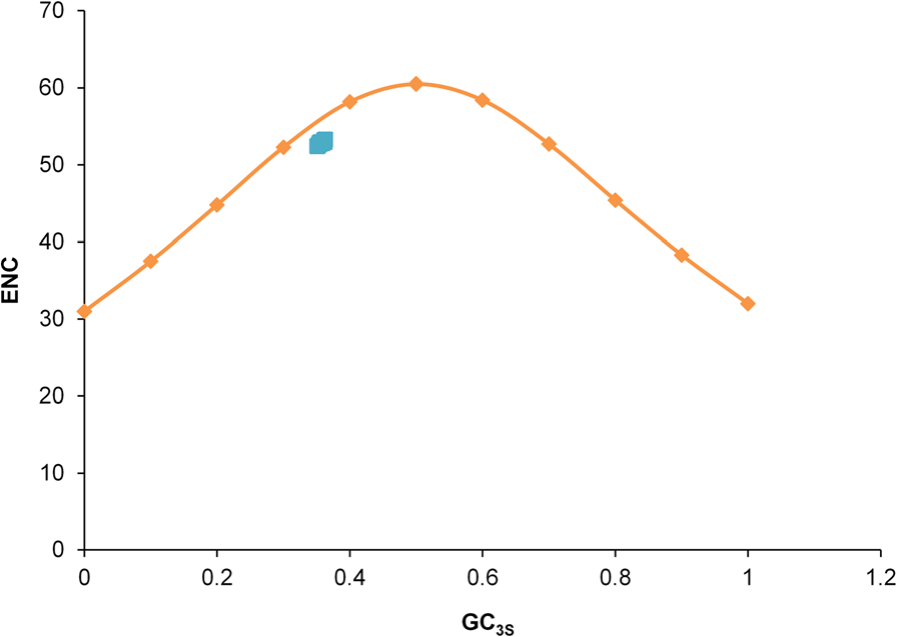
The relationship between the effective number of codons (ENC) values and GC content at the third synonymous codon position (GC_3S_). The curve indicates the expected codon usage if GC compositional constraints alone account for codon usage bias

To determine whether the patterns of codon usage have been influenced by mutation pressure, ENC-plot, correlation, linear regression and neutrality plot analyses were performed. In case of ENC-plot, The GC_3S_ values were plotted against ENC, which showed that all spots clustered slightly below on the left side of the expected curve (Fig. 3). This indicates that mutational pressure has dominated in shaping codon usage patterns of MARVs.

In the next step, correlation analysis among the nucleotide compositions, codon compositions, and ENC values was performed (Table 3). Several strong and significant correlations (*P <* 0*.*01; *P <* 0*.*05) were observed between nucleotide compositions and codon compositions. GC and GC_1,2S_ were also compared with GC_3S_ and highly significant positive correlations (GC_1,2S_ versus GC_3S_: *r =* 0*.*747, *P <* 0*.*01; GC versus GC_3S_: *r =* 0*.*828, *P <* 0*.*01) were observed. Furthermore, a significant positive correlation between GC_3S_ and ENC values (*r =* 0.424, *P <* 0*.*001) as well as a significantly negative correlation between AU_3S_ and ENC (*r = −*0.868, *P <* 0*.*001) were also observed. These results indicate that compositional constraints under mutation pressure have shaped the codon usage pattern for MARV.

**Table 3:** Summary of correlation analysis between nucleotide constraints in MARV genomes

	A_3S_ %	U_3S_ %	C_3S_ %	G_3S _%	GC_3S_ %	AU_3S_ %
A %	0.818**	0.321*	−0.373**	−0.773**	−0.659**	0.688**
U %	0.260*	0.775**	−0.773**	−0.334**	−0.641**	0.604**
C %	−0.571**	−0.819**	0.911**	0.623**	0.865**	−0.851**
G %	−0.640**	−0.231^NS^	0.255*	−0.673**	0.535**	−0.561**
GC %	−0.755**	−0.752**	0.774**	0.831**	0.929**	−0.915**
AU %	0.712**	0.723**	−0.798**	0.745**	−0.892**	0.886**

The numbers in the each column represent correlation coefficient “*r*” values, which are calculated in each correlation analysis

*NS* non-significant (*P* > 0.05)

*represents 0.01 < *P* < 0.05

**represents *P* < 0.01

In addition to correlation analysis, linear regression analysis was also performed to determine correlations between *f'*_1_ and *f'*_2_ and nucleotide constraints of MARV genomes (Table 4). In agreement to above findings, significant correlations were observed between both axes and compositional quantities indicating that mutation pressure has played a major role in shaping the dynamics of codon usage patterns within MARV genomes.

**Table 4:** Summary of correlation between the first two principle axes and nucleotide constraints in MARV genomes

Base composition	*f'*_1_	*f'*_2_
A_3S_ %	0.373**	−0.384**
U_3S_ %	0.548**	−0.046**
C_3S _%	−0.543**	0.103^NS^
G_3S_ %	−0.382**	0.492**
GC_3S_ %	−0.456**	0.361**
GC %	−0.406**	0.388**
A %	0.58^NS^	−0.585**
U %	0.610**	0.163^NS^
G %	0.74^NS^	0.764**
C %	−0.589**	0.099 ^NS^
AU %	0.405**	−0.316*
AU_3S_ %	0.462**	−0.337**

The numbers in the each column represents correlation coefficient “*r*” values, which are calculated in each correlation analysis

*NS* non-significant (*P* > 0.05)

*represents 0.01 < *P* < 0.05

**represents *P* < 0.01

**Fig. 4:**
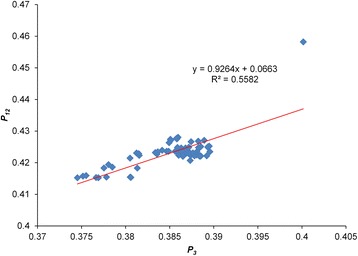
Neutrality plot analysis of the GC_1,2S_ and that of the third codon position (GC_3S_). GC_1,2S_ stands for the average value of GC content in the first and second position of the codons (GC_1S_ and GC_2S_). While GC_3S_ refers to the GC content in the third position. The solid red line is the linear regression of GC_1,2S_ against GC_3S_

A neutrality plot was constructed between *P*_12_ (GC_1,2S_) and *P*_3_ (GC_3S_) values to determine the extent of variation between mutation pressure and natural selection (Fig. 4). A significant positive correlation (*r =* 0*.*747, *P <* 0*.*01) was found between *P*_12_ and *P*_3_ values with a correlation coefficient of 0.926 ± 0.067, suggesting that the effect of directional mutation pressure is present at all codon positions. The correlation coefficient showed that the relative neutrality is 92.6 % or that the relative constraint of GC_3S_ (100 % neutrality or 0 % constraint) is only 7.4 %, thereby showing that mutation pressure is dominant over natural selection in shaping codon usage bias of MARV.

### Natural selection is a minor player in shaping MARV codon usage patterns

To determine the potential influence of natural selection, linear regression analysis was performed between General average hydropathicity (GRAVY) and aromaticity (ARO) values and the *f'*_1_, *f'*_2_, ENC, GC, and GC_3S_ values to investigate the influence of natural selection on MARV codon usage patterns. The correlations of both GRAVY and ARO with *f'*_1_ were non-significant, whereas GRAVY and ARO showed significant positive and negative correlations with *f'*_2_, respectively. Furthermore, it was found that GRAVY had significantly positive correlations with ENC, GC_3S_, and GC values, whereas ARO had significant negative correlations with GC_3S_ and GC and non-significant correlations with the ENC value (Table 5). The non-significant correlation of both GRAVY and ARO with *f'*_1_, which accounts for 65.55 % of the total variation, shows that natural selection has contributed to some extent; however, it is not the most substantial influencing factor on MARV codon usage patterns.

**Table 5:** Correlation analysis among GRAVY, ARO, ENC, GC_3S_, GC, and the first two principle axes

		*f'*_1_	*f'*_2_	ENC	GC_3S_	GC
GRAVY	*r*	−0.015^NS^	0.635**	0.594**	0.544**	0.480**
	*P*	0.907	0.000	0.000	0.000	0.000
ARO	*r*	0.018^NS^	−0.596**	−0.226 ^NS^	−0.283*	−0.312*
	*P*	0.887	0.000	0.075	0.025	0.013

*ARO* aromaticity; *NS* non-significant (*P* > 0.05)

*represents 0.01 < *P* < 0.05

**represents *P* < 0.01

### Codon usage adaptation in MARV

In order to determine codon usage optimization and adaptation of MARV to its hosts, CAI analysis was performed. A mean CAI of 0.712 was obtained for MARV genes in relation to *H. sapiens*, while a mean CAI of 0.534 was obtained in relation to *R. aegyptiacus* (Additional file 3: Table S2). There was a trend for a lower CAI values for MARV in relation to *R. aegyptiacus*, with the consequent lower efficiency of protein synthesis in *R. aegyptiacus*. Furthermore, correlation was investigated between CAI and ENC values to examine the relative influence of mutation pressure and natural selection. The CAI values of MARV genes in relation to *H. sapiens* and *R. aegyptiacus* were found to be negatively (*r* = −0.423, *P <* 0*.*001) and positively (*r* = 0.314, *P =* 0*.*009) correlated with ENC values respectively. This phenomenon reflected that the interplay of codon usage between MARV and its hosts have influenced viral fitness, survival and evolution which implies influence of natural selection on MARV.

### Dinucleotide abundance has a minor influence on MARV codon usage patterns

To study the possible effect of dinucleotides on codon usage, we calculated the relative abundances of the 16 dinucleotides from the MARV coding sequences. The occurrence of dinucleotides was found to be non-random, and only CpU was present at the expected frequencies (i.e., 1.0) (Table 6). Furthermore, only CpA was over-represented and showed marginal over-representation (1.23 ± 0.01). CpG (mean ± SD = 0.51 ± 0.01) and GpC (mean ± SD = 0.90 ± 0.03) were both under-represented. The analysis of RSCU values of both CpG-containing codons (CCG, GCG, UCG, ACG, CGC, CGG, CGU, and CGA) and GpC-containing codons (GCU, GCC, GCA, UGC, AGC, and GGC) showed that all codons were also under-represented (RSCU < 0.6) and were not preferred codons for their respective amino acids (Table 2). Similar to CpG and GpC, the relative abundance of UpA also deviated from the “normal range” (mean ± SD = 0.69 ± 0.01) and was under-represented. Except for UUA (RSCU = 1.72), which is a preferred codon for the amino acid leucine, the remaining five UpA containing codons (CUA, GUA, UAU, UAC, and AUA) were under-represented (RSCU < 0.6) and not preferred codons. Five (UCA, ACA, GCA, CAA, and CAU) out of eight codons that contain CpA (CCA, CAG, and CAC) were also over-represented and preferred codons compared with the rest of the codons for their respective amino acids (Table 2). Correlation between the relative abundance of dinucleotides with the *f'*_1_ and *f'*_2_ was also investigated. Fourteen and 12 out of 16 dinucleotides showed significant positive and negative correlations with the *f'*_1_ and *f'*_2_, respectively (Table 6).

**Table 6:** Summary of correlation analysis between the first two principal axes and relative abundance of dinucleotides in MARV genomes

		UU	UC	UA	UG	CU	CC	CA	CG
Mean ± SD		0.32 ± 0.00	1.11 ± 0.01	0.69 ± 0.01	1.17 ± 0.01	1.00 ± 0.03	1.05 ± 0.02	1.23 ± 0.01	0.51 ± 0.01
Range		0.31–.32	1.07–0.13	0.68–0.71	1.16–1.20	0.97–1.07	1.03–1.10	1.21–1.25	0.49–0.53
*f'*_1_	*r*	−0.552**	0.104^NS^	0.320*	0.003^NS^	0.517**	−0.348**	−0.326**	−0.394**
	*P*	0.000	0.415	0.011	0.979	0.000	0.005	0.009	0.001
*f'*_2_	*r*	−0.603**	−0.040^NS^	−0.146^NS^	0.533**	−0.185^NS^	0.318*	−0.397**	−0.626**
	*P*	0.000	0.758	0.252	0.000	0.147	0.011	0.001	0.000
		AU	AC	AA	AG	GU	GC	GA	GG
Mean ± SD		0.97 ± 0.01	0.91 ± 0.01	1.06 ± 0.01	1.05 ± 0.02	0.82 ± 0.02	0.90 ± 0.03	1.11 ± 0.01	1.21 ± 0.03
Range		0.96–0.99	0.89–0.93	1.04–1.07	1.03–1.11	0.79–0.86	0.86–0.97	1.08–1.14	1.16–1.24
*f'*_1_	*r*	−0.420**	−0.455**	−0.412**	0.681**	−0.369**	−0.735**	0.420**	−0.558**
	*P*	0.001	0.000	0.001	0.000	0.003	0.000	0.001	0.000
*f'*_2_	*r*	−0.515**	−0.550**	0.192^NS^	0.238^NS^	0.357**	0.265*	−0.291*	−0.788**
	*P*	0.000	0.000	0.132	0.060	0.004	0.036	0.021	0.000

*NS* non-significant (*P* > 0.05)

*represents 0.01 < *P* < 0.05

**represents *P* < 0.01

### MARV codon usage patterns are antagonist toward its hosts

To determine the influence of host on MARV codon usage patterns, the codon usage of MARV isolates was compared with that of its two hosts, *H. sapiens* and *R. aegyptiacus*, via comparison of RSCU values. The results showed that the codon usage patterns and selection of preferred codons in MARV genomes is antagonist to both *H. sapiens* and *R. aegyptiacus* for majority of codons (Table 2). The only exception was codon AGA, which was a preferred codon for the amino acid arginine in MARV and *H. sapiens* but not in *R. aegyptiacus*. Moreover, analysis of RSCU values in a heatmap also showed that the MARV RSCU values did not cluster along any of its hosts RSCU values (Additional file 4: Figure S2).

### Selection pressure by *R. aegyptiacus* is stronger compared with that of *H. sapiens* on MARV’s overall codon usage patterns

**Fig. 5:**
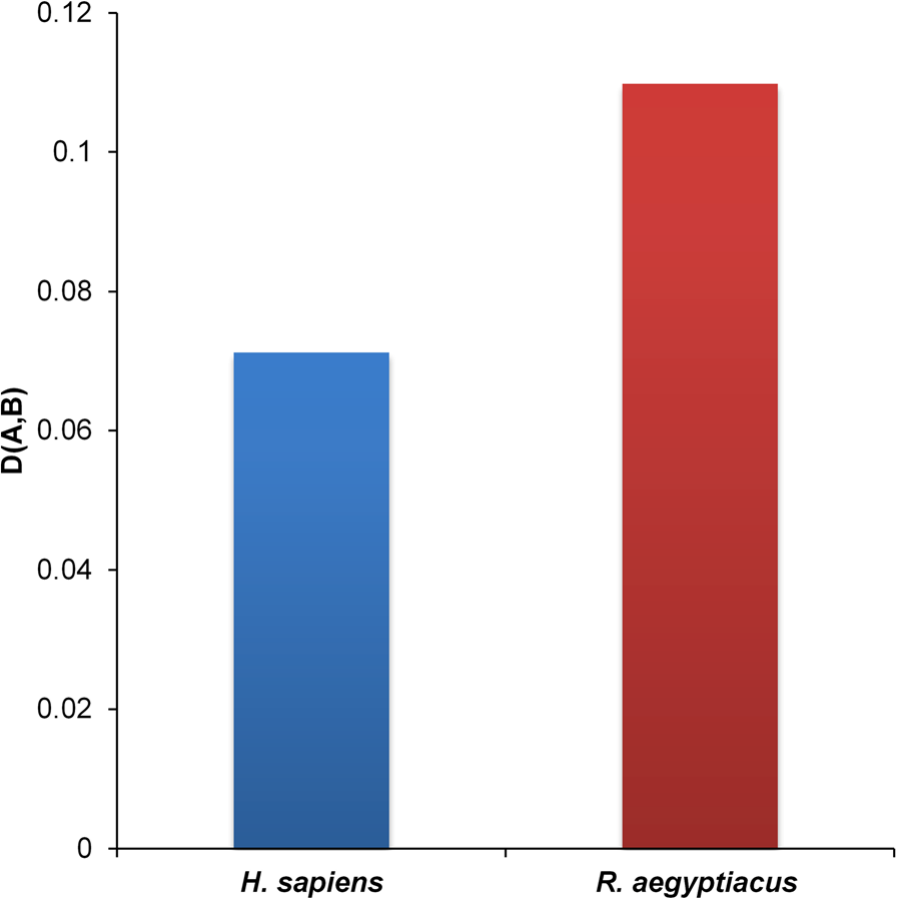
Similarity index analysis of the codon usage between MARV and its hosts

To determine how the overall codon usages of MARV’s hosts have contributed to evolution of virus codon usage patterns, similarity index analysis was conducted. It was found that *R. aegyptiacus* exerted a more dominant effect on shaping MARV codon usage compared with that of *H. sapiens*, as the similarity index was found to be higher in *R. aegyptiacus* (Fig. 5).

## Discussion

In the present study, we analyzed synonymous codon usage in coding sequences from 63 MARV genomes to understand its molecular evolution under the influence of multiple viral, host, and environmental factors. It has previously been shown that codon usage bias, or preference for one type of codon over another, can be greatly influenced by overall genomic composition [42]. Nucleotide composition analysis showed that A and U nucleotides constitute the majority of overall nucleotide composition in MARV genomes (Table 1). The RSCU analysis also showed that MARV genomes exhibit greater codon usage bias toward A- and U-ended codons (Table 2). Therefore, once it is established that there is codon bias toward A- and U-ended codons in MARV genomes, we next determined the extent of this bias within and in between different MARV isolates. This was accomplished by ENC analyses. In the case of MARV, the mean ENC value was found to be 54.2 in MARV coding sequences, which indicates slightly biased, relatively stable and conserved genomic composition among different MARV genomes. Studies have shown that ENC and gene expression are inversely correlated with each other; e.g., a lower ENC value indicates a higher codon usage preference and higher gene expression. Overall, it was found that the codon usage bias and gene expression among different MARV genomes is similar and is slightly biased. The low codon usage bias has also been observed among other RNA viruses, such as the EBOV (ENC: 57.23) [43], chikungunya virus (CHIKV; ENC: 55.56) [44], bovine viral diarrhea virus (ENC: 50.91) [45], classical swine fever virus (ENC: 51.7) [13], hepatitis C virus (HCV; ENC: 52.62) [46], and West Nile virus (ENC: 53.81) [11]. A possible explanation given for this is that the low codon bias of RNA viruses might be advantageous for efficient replication in host cells by reducing the synthesis machinery competition between the virus and host with potentially distinct codon preferences. Whether the same holds true for MARVs as well, warrants further investigations. However, this can be attributed to the fact that MARVs maintain low yet surviving replication rate within in its natural host, *R. aegyptiacus* without causing any disease conditions [47]. Therefore, it seems that evolution of low codon bias within MARV coding sequences have enabled it to successfully maintain its survival cycle within both of its hosts each of which possess distinct codon usage preferences from that of MARV (Table 2).

Considering the multivariate nature of codon usage, we next performed PCA analysis on RSCU values to determine the trends of codon usage variations that showed that *f'*_1_ accounted for the major portion of codon usage variation followed by *f'*_2_. Moreover, MARV isolates formed three separate PCA clusters following distribution on principal axes. The clustering of diverse MARV lineages that are separated by thousands of miles within a single cluster as well as clustering of closely related lineages into different clusters highlights an important role of the mobility of MARV’s natural host, *R. aegyptiacus*. This also indicates that the isolates of MARV might have independently evolved in three clusters after diverging from a common ancestor that potentially originated from DRC, based on inclusion of DRC isolates in all three clusters. Moreover, it appears that the geographical diversity and associated factors, such as presence of natural host within region of infection, climatic features, and host susceptibility, have contributed to shaping codon usage in MARV genomes.

Our initial analysis indicated influence of nucleotide constraints on MARV codon usage patterns. However, it has previously been shown that, although overall RSCU could reveal the codon usage pattern for genomes, it may hide the codon usage variation among different genes in a genome [48], thereby indicating that composition frequencies of nucleotides are not always the only factor associated with codon usage patterns. The ENC plot is widely used to determine codon usage variation among genes in different organisms. It has been postulated that the ENC-plot of genes for which codon choice is constrained only by compositional constraints or mutation pressure will lie on the continuous curve of the predicted ENC values [32]. When the ENC and GC_3S_ values of MARVs were plotted, it was found that although none of the isolates fell on the expected continuous curve but clustered closely below the curve, thereby showing major influence of mutation pressure on MARV codon usage patterns and of natural selection to some extent. Besides that, it has also been reported previously that both mutation pressure and natural selection can influence the overall ENC and it might not be a robust index to show the relative contribution of mutation and selection on structuring codon usage patterns. Moreover, the codon usage bias of base composition of the genes of a species with A/U biased genomes will behave differentially than those species with G/C biased genomes, as such, ENC-GC_3S_ plot might be potentially misleading. In contrast, CAI is suggested as the most robust index for showing the influence of natural selection on codon usage patterns of such genes [27, 30, 31, 49]. CAI is regarded as a measure of gene expression and can be used to assess the adaptation of viral genes to their hosts. It has been postulated that the highly expressed genes exhibit a stronger bias for particular codons compared with genes that are less expressed. Compared with ENC, which is another way of calculating codon usage bias and measures deviation from a uniform bias (null hypothesis), CAI measures the deviation of a given protein coding gene sequence with respect to a reference set of genes [27]. If CAI value is high, then codon usage bias is extremely high and the influence of natural selection is prevailing. CAI values were calculated for MARV genes separately for both of its hosts. MARV genes showed higher CAI values for *H. sapiens* (0.712) as compared to *R. aegyptiacus* (0.534) indicating that natural selection from both hosts have influenced the codon usage patterns of MARV. Furthermore, comparative analysis of CAI values between MARV and its hosts suggests that MARV genes have optimized their codon usage patterns to utilize the translational resources of *H. sapiens* more efficiently than that of *R. aegyptiacus*. A higher CAI values of MARV genes for *H. sapiens* represents an interesting evolutionary step which might have supported MARV to turn out to be a highly pathogenic virus for *H. sapiens* and at the same time remaining completely harmless for its natural host, *R. aegyptiacus*.

Among multiple influencing factors, mutation pressure and natural selection are considered the two major factors that shape codon usage patterns [50]. A general mutation pressure that affects the whole genome would certainly account for the majority of the codon usage among certain RNA viruses [42]. The ENC and CAI analyses highlighted the influence of both mutation pressure and natural selection on codon usage patterns of MARV genes. In order to determine the share of each factor on evolution of MARV codon usage patterns, a neutrality plot analysis was performed which showed that influence of mutation pressure dominates over natural selection. Furthermore, we also examined the influence of mutation pressure on MARV codon usage via correlation and linear regression analyses between different nucleotide compositional constraints, ENC, and principal axes. Strong and significant correlations were observed, which indicates a dominant influence of mutation pressure. This was further supported when these indices were plotted against the first two principal axes via PCA, and significant strong correlations were observed. However, in the case of MARV genomes, involvement of factors other than mutation pressure such as natural selection cannot be ignored because nucleotide base compositions showed variation, distribution of MARV isolates were although close to but still below the expected curve on ENC plot, and there was a weak codon bias. A weak codon usage bias may be caused by natural selection when the viruses try to adapt to the host cell [51–53]. It has been suggested that, if synonymous codon usage bias is affected by mutation pressure alone, then the frequency of nucleotides A and U/T should be equal to that of C and G at the synonymous codon third position [53]. To test this phenomena in MARV, PR2 biasness plot analysis was performed on four fold degenerate codons. The occurrence frequency of AU and GC nucleotides at the synonymous codon third position was not found to be equal and AU biased preference was observed in four fold degenerate codons of MARV genes which indicates the potential influence of natural selection on codon usage patterns of MARV genes. In addition to this, correlation analysis between principal axes and GRAVY and ARO also revealed that, although natural selection has influenced MARV codon usage patterns to some extent, it is much weaker compared with mutation pressure.

Dinucleotide abundance has been reported to influence overall codon usage bias in several organisms, including DNA and RNA viruses [37, 54, 55]. Toll-like receptor 9 (TLR9), which is a type of intracellular pattern recognition receptor (PRR), recognizes unmethylated CpGs, which leads to activation of several immune response pathways [56]. The vertebrate immune system relies on unmethylated CpG recognition in DNA molecules as a signature of infection, and CpG under-representation in RNA viruses is exclusively observed in vertebrate viruses; therefore, it is reasonable to suggest that a TLR9-like mechanism exists in the vertebrate immune system that recognizes CpGs when in an RNA context (such as in the genomes of RNA viruses) and triggers immune responses [57]. In contrast to the CpG usage of + ssRNA viruses that are greatly influenced by their hosts and because of which + ssRNA viruses mimic their hosts’ CpG usage, -ssRNA viruses do not produce DNA intermediates during the replication of their genome. As a result, CpGs are under-represented, independent of the infected host or their phylogenetic relationship. The under-representation of CpG in -ssRNA viruses is therefore due to the U/A mutation bias in overall genomic composition that further indicates a dominating effect of mutation pressure [54, 58]. In the case of MARV, none of the dinucleotides were found at the expected frequencies and were also markedly under-represented. As inferred from the RSCU analysis, codons containing CpG and UpA dinucleotides were also under-represented and were not preferred codons for their respective amino acids within MARV genomes. These results collectively indicate that, although dinucleotide representation has some influence over the codon usage of MARVs, the overall influence is not strong because of exceptions among dinucleotide frequencies and selection of preferred codons in MARV genomes.

It has been previously shown that, among many other factors, the codon usage patterns of viruses are also affected by its hosts [59]. For example, the codon usage pattern of poliovirus is reported to be mostly coincident with that of its host [60], whereas the codon usage pattern of hepatitis A was reported to be antagonistic to that of its host [61]. Alternatively, some viruses exhibit a mix of both coincidence and antagonism, such as the HCV [46], enterovirus 71 [9], and CHIKV [44]. However, MARV showed almost complete antagonism to both of its hosts, as inferred from the RSCU analysis with the exception for a common preferred codon for arginine between MARV and *H. sapiens* (Table 2). A recent codon usage analysis in the EBOV, which is also from the same family as MARV and spread via bats, reported similar antagonism of RSCU toward its host, *H. sapiens* [43]. It has been proposed that the coincident portions of codon usage among viruses and their hosts could enable the corresponding amino acids to be efficiently translated, whereas the antagonistic portions of codon usage may enable viral proteins to be properly folded, although the translation efficiency of the corresponding amino acids might decrease [46]. Whether the same holds true for MARV warrants further investigations. In addition to the RSCU comparison analysis, we also performed a similarity index analysis to determine which of the MARV hosts have a dominant influence over its RSCU patterns. Evidence of selection pressure from both hosts was detected which is in agreement to the CAI analysis; however, level of pressure was significantly different. Compared with *H. sapiens*, *R. aegyptiacus* have a more profound effect on shaping MARV RSCU patterns, as inferred from the similarity index analysis. As *R. aegyptiacus* is consider as the natural reservoir and host for MARV, it makes sense that virus has evolved its genomic features to a stable level in order to better adapt to its primary host’s environment. It has also been recently suggested that flight, a factor common to all bats but to no other mammals, provides an intensive selective force for coexistence with viral parasites through a daily cycle that elevates metabolism and body temperature analogous to the febrile response in other mammals. On an evolutionary scale, this host-virus interaction might have resulted in the large diversity of zoonotic viruses in bats, possibly through bat viruses adapting to be more tolerant of the fever response and less virulent to their natural hosts [47].

## Conclusions

In summary, this study showed that overall codon usage bias within MARVs is slightly biased, and the major factor that has contributed to shaping codon usage is mutation pressure followed by influence of hosts. In addition, natural selection, environment, geographical conditions, and dinucleotides have also been determined to influence codon usage. The evolution of MARV probably reflects a dynamic process of mutation and natural selection to adapt its codon usage to different environments and hosts.

## Availability of supporting data

The data sets supporting the results of this article are available in the Dryad digital repository http://dx.doi.org/10.5061/dryad.3hc5t.

## Additional files

Additional file 1: Table S1. Demographics of MARV genomes that were analyzed in the present study. (DOCX 23 kb) Additional file 2: Figure S1. Comparative analysis of relative synonymous codon usage (RSCU) patterns between MARV, *H. sapiens*, and *R. aegyptiacus*. (TIFF 2285 kb) Additional file 3: Table S2. Codon adaptation index (CAI) analysis of MARV coding sequences. (XLSX 10 kb) Additional file 4: Figure S2. Heatmap-based representation of clustering of RSCU values of MARV, *H. sapiens*, and *R. aegyptiacus*. (TIFF 101 kb)
