# Supplementary material for: Genomic analysis of codon usage shows influence of mutation pressure, natural selection, and host features on Marburg virus evolution
Source: BMC Evol Biol. 2015 Aug 26;15:174. doi: 10.1186/s12862-015-0456-4 (PMC4550055; doi:10.1186/s12862-015-0456-4)
Supplement: Additional file 1: Table S1. — Demographics of MARV genomes that were analyzed in the present study. (DOCX 23 kb) [file 12862_2015_456_MOESM1_ESM.docx]

**Table S1:** Demographics of MARV genomes analyzed in present study.

| **S. No** | **Strain Name** | **GenBank Accession** | **Length (bp)** | **Year** | **Host** | **Country** |
| --- | --- | --- | --- | --- | --- | --- |
| 1 | Germany_ Marburg | EF446132 | 19112 | 1967 | - | Germany |
| 2 | Kenya | EF446131 | 19114 | 1987 | - | Kenya |
| 3 | R1 | EU500827 | 19114 | 1987 | *H. sapiens* | Kenya |
| 4 | R2 | EU500828 | 19114 | 1987 | - | Kenya |
| 5 | R3 | EU500826 | 19114 | 1987 | - | Kenya |
| 6 | Ravn | DQ447649 | 19114 | 1987 | *H. sapiens* | Kenya |
| 7 | 05DRC99 | DQ447651 | 19114 | 1999 | *H. sapiens* | DRC |
| 8 | 07DRC99 | DQ447650 | 19114 | 1999 | *H. sapiens* | DRC |
| 9 | 09DRC99 | DQ447652 | 19114 | 1999 | *H. sapiens* | DRC |
| 10 | MARV/01 DRC | JX458833 | 19114 | 1999 | *H. sapiens* | DRC |
| 11 | MARV/02 DRC | JX458851 | 19114 | 1999 | *H. sapiens* | DRC |
| 12 | MARV/03 DRC | JX458826 | 19113 | 1999 | *H. sapiens* | DRC |
| 13 | MARV/04 DRC | JX458825 | 19113 | 1999 | *H. sapiens* | DRC |
| 14 | MARV/06 DRC | JX458830 | 19114 | 1999 | *H. sapiens* | DRC |
| 15 | MARV/12 DRC | JX458840 | 19114 | 2000 | *H. sapiens* | DRC |
| 16 | MARV/13 DRC | JX458845 | 19114 | 2000 | *H. sapiens* | DRC |
| 17 | MARV/14 DRC | JX458831 | 19114 | 2000 | *H. sapiens* | DRC |
| 18 | MARV/15 DRC | JX458847 | 19114 | 2000 | *H. sapiens* | DRC |
| 19 | MARV/16 DRC | JX458839 | 19114 | 2000 | *H. sapiens* | DRC |
| 20 | MARV/17 DRC | JX458838 | 19114 | 2000 | *H. sapiens* | DRC |
| 21 | MARV/18 DRC | JX458842 | 19114 | 2000 | *H. sapiens* | DRC |
| 22 | MARV/19 DRC | JX458828 | 19114 | 2000 | *H. sapiens* | DRC |
| 23 | MARV/20 DRC | JX458841 | 19114 | 2000 | *H. sapiens* | DRC |
| 24 | MARV/21 DRC | JX458843 | 19114 | 2000 | *H. sapiens* | DRC |
| 25 | MARV/22 DRC | JX458848 | 19114 | 2000 | *H. sapiens* | DRC |
| 26 | MARV/23 DRC | JX458835 | 19114 | 2000 | *H. sapiens* | DRC |
| 27 | MARV/24 DRC | JX458834 | 19114 | 2000 | *H. sapiens* | DRC |
| 28 | MARV/25 DRC | JX458849 | 19114 | 2000 | *H. sapiens* | DRC |
| 29 | MARV/26 DRC | JX458844 | 19114 | 2000 | *H. sapiens* | DRC |
| 30 | MARV/27 DRC | JX458837 | 19114 | 2000 | *H. sapiens* | DRC |
| 31 | MARV/28 DRC | JX458846 | 19114 | 2000 | *H. sapiens* | DRC |
| 32 | MARV/29 DRC | JX458836 | 19114 | 2000 | *H. sapiens* | DRC |
| 33 | MARV/30 DRC | JX458832 | 19114 | 2000 | *H. sapiens* | DRC |
| 34 | MARV/32 DRC | JX458827 | 19114 | 2000 | *H. sapiens* | DRC |
| 35 | MARV/33 DRC | JX458850 | 19114 | 2000 | *H. sapiens* | DRC |
| 36 | MARV/34 DRC | JX458829 | 19114 | 2000 | *H. sapiens* | DRC |
| 37 | Ang0126 | DQ447656 | 19114 | 2005 | *H. sapiens* | Angola |
| 38 | Ang0214 | DQ447657 | 19114 | 2005 | *H. sapiens* | Angola |
| 39 | Ang0215 | DQ447658 | 19114 | 2005 | *H. sapiens* | Angola |
| 40 | Ang0754 | DQ447659 | 19114 | 2005 | *H. sapiens* | Angola |
| 41 | Ang0998 | DQ447660 | 19114 | 2005 | *H. sapiens* | Angola |
| 42 | Ang1379c | DQ447653 | 19114 | 2005 | *H. sapiens* | Angola |
| 43 | Ang1381 | DQ447654 | 19114 | 2005 | *H. sapiens* | Angola |
| 44 | Ang1386 | DQ447655 | 19114 | 2005 | *H. sapiens* | Angola |
| 45 | Uganda 01Uga07 | FJ750957 | 19114 | 2007 | *H. sapiens* | Uganda |
| 46 | Uganda 02Uga07 | FJ750953 | 19114 | 2007 | *H. sapiens* | Uganda |
| 47 | Uganda 188Bat2007 | FJ750955 | 19114 | 2007 | *R. aegyptiacus* | Uganda |
| 48 | Uganda 331Bat2007 | FJ750959 | 19114 | 2007 | *R. aegyptiacus* | Uganda |
| 49 | Uganda 371Bat2007 | FJ750958 | 19114 | 2007 | *R. aegyptiacus* | Uganda |
| 50 | Uganda 44Bat2007 | FJ750954 | 19114 | 2007 | *R. aegyptiacus* | Uganda |
| 51 | Leiden | JN408064 | 19113 | 2008 | *H. sapiens* | Netherlands |
| 52 | MARV/164/Qbat | JX458853 | 19114 | 2008 | *R. aegyptiacus* | Uganda |
| 53 | MARV/53 Qbat | JX458852 | 19114 | 2008 | *R. aegyptiacus* | Uganda |
| 54 | MARV/982 | FJ750956 | 19114 | 2008 | *R. aegyptiacus* | Uganda |
| 55 | MARV/1175/Qbat | JX458854 | 19114 | 2009 | *R. aegyptiacus* | Uganda |
| 56 | MARV/1328/Qbat | JX458858 | 19114 | 2009 | *R. aegyptiacus* | Uganda |
| 57 | MARV/843/Qbat | JX458855 | 19114 | 2009 | *R. aegyptiacus* | Uganda |
| 58 | MARV/914/Qbat | JX458856 | 19114 | 2009 | *R. aegyptiacus* | Uganda |
| 59 | RAVV/1304/Qbat | JX458857 | 19114 | 2009 | *R. aegyptiacus* | Uganda |
| 60 | Mbg-422-2012 | KC545387 | 19114 | 2012 | *H. sapiens* | Uganda |
| 61 | Mbg-423-2012 | KC545388 | 19114 | 2012 | *H. sapiens* | Uganda |
| 62 | Musoke | DQ217792 | 19111 | - | - | Kenya |
| 63 | Ozolin | AY358025 | 19151 | 1975 | - | South Africa |

Dashes (-) indicates data not available.

DRC: Demographic Republic of Congo
