# Supplementary figures and images for: Genomic analysis of codon usage shows influence of mutation pressure, natural selection, and host features on Marburg virus evolution
Source: BMC Evol Biol. 2015 Aug 26;15:174. doi: 10.1186/s12862-015-0456-4 (PMC4550055; doi:10.1186/s12862-015-0456-4)

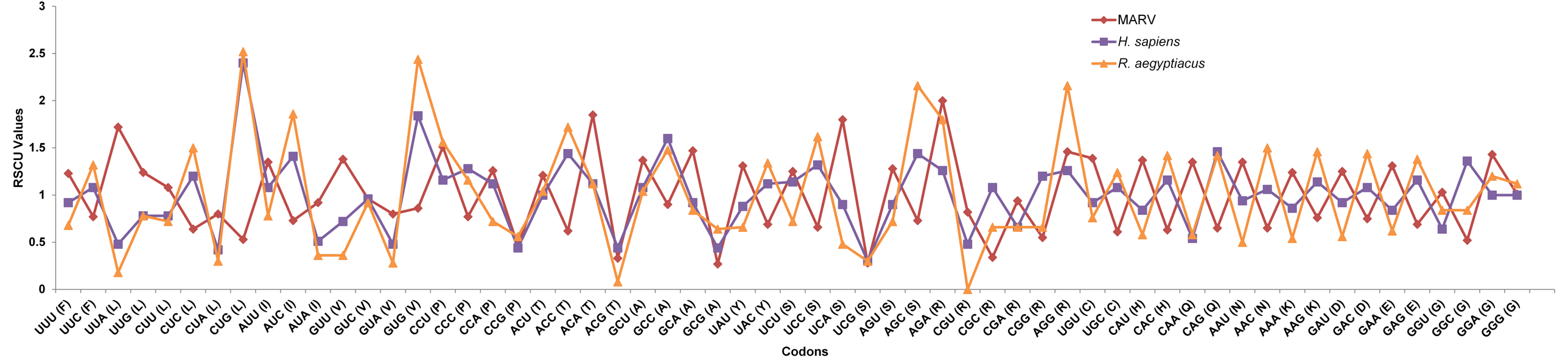

Supplement: Additional file 2: Figure S1. — Comparative analysis of relative synonymous codon usage (RSCU) patterns between MARV, H. sapiens, and R. aegyptiacus. (TIFF 2285 kb) [file 12862_2015_456_MOESM2_ESM.tiff]

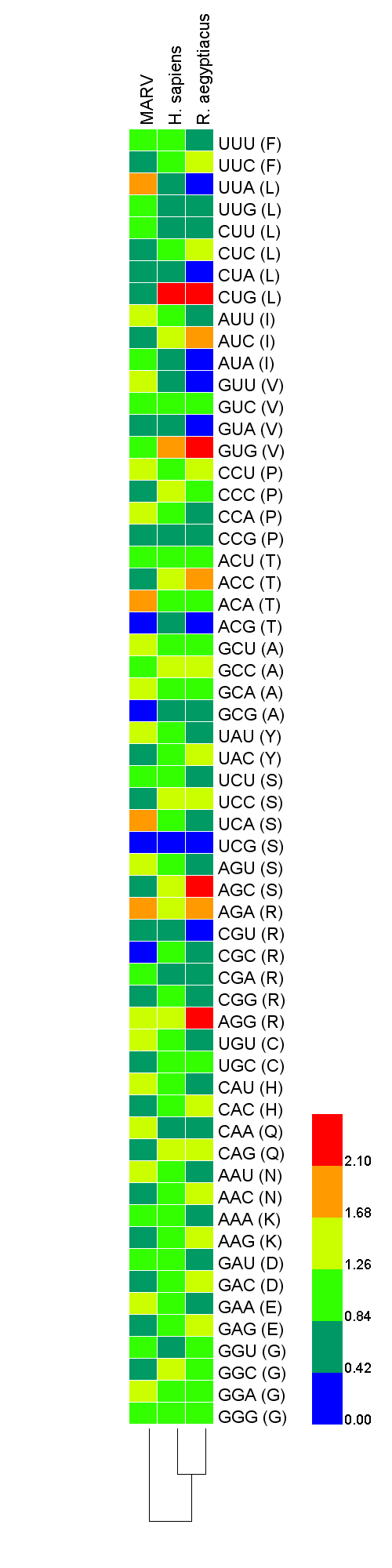

Supplement: Additional file 4: Figure S2. — Heatmap-based representation of clustering of RSCU values of MARV, H. sapiens, and R. aegyptiacus. (TIFF 101 kb) [file 12862_2015_456_MOESM4_ESM.tiff]
